# The Octadecaneuropeptide ODN Protects Astrocytes against Hydrogen Peroxide-Induced Apoptosis via a PKA/MAPK-Dependent Mechanism

**DOI:** 10.1371/journal.pone.0042498

**Published:** 2012-08-21

**Authors:** Yosra Hamdi, Hadhemi Kaddour, David Vaudry, Seyma Bahdoudi, Salma Douiri, Jérôme Leprince, Helene Castel, Hubert Vaudry, Marie-Christine Tonon, Mohamed Amri, Olfa Masmoudi-Kouki

**Affiliations:** 1 Laboratory of Functional Neurophysiology and Pathology, Research Unit UR/11ES09, Department of Biological Sciences, Faculty of Science of Tunis, University Tunis El Manar, Tunis, Tunisia; 2 Inserm U982, Laboratory of Neuronal and Neuroendocrine Communication and Differentiation, University of Rouen, Mont-Saint-Aignan, France; 3 International Associated Laboratory Samuel de Champlain, Mont-Saint-Aignan, France; 4 Regional Platform for Cell Imaging of Haute-Normandie (PRIMACEN), Institute for Medical Research and Innovation (IRIB), University of Rouen, Mont-Saint-Aignan, France; University of Cordoba, Spain

## Abstract

Astrocytes synthesize and release endozepines, a family of regulatory peptides, including the octadecaneuropeptide (ODN) an endogenous ligand of both central-type benzodiazepine (CBR) and metabotropic receptors. We have recently shown that ODN exerts a protective effect against hydrogen peroxide (H_2_O_2_)-induced oxidative stress in astrocytes. The purpose of the present study was to determine the type of receptor and the transduction pathways involved in the protective effect of ODN in cultured rat astrocytes. We have first observed a protective activity of ODN at very low concentrations that was abrogated by the metabotropic ODN receptor antagonist cyclo_1–8_[DLeu^5^]OP, but not by the CBR antagonist flumazenil. We have also found that the metabotropic ODN receptor is positively coupled to adenylyl cyclase in astrocytes and that the glioprotective action of ODN upon H_2_O_2_-induced astrocyte death is PKA- and MEK-dependent, but PLC/PKC-independent. Downstream of PKA, ODN induced ERK phosphorylation, which in turn activated the expression of the anti-apoptotic gene Bcl-2 and blocked the stimulation by H_2_O_2_ of the pro-apoptotic gene Bax. The effect of ODN on the Bax/Bcl-2 balance contributed to abolish the deleterious action of H_2_O_2_ on mitochondrial membrane integrity and caspase-3 activation. Finally, the inhibitory effect of ODN on caspase-3 activity was shown to be PKA and MEK-dependent. In conclusion, the present results demonstrate that the potent glioprotective action of ODN against oxidative stress involves the metabotropic ODN receptor coupled to the PKA/ERK-kinase pathway to inhibit caspase-3 activation.

## Introduction

Diazepam-binding inhibitor (DBI) is an 86-amino acid polypeptide that has been originally isolated from rat brain extracts as an endogenous ligand of benzodiazepine receptors [1]. DBI and its derived peptides, including the octadecaneuropeptide ODN, are collectively termed endozepines [2], [3]. It was initially reported that the endozepines ODN and DBI act as inverse agonists of central-type benzodiazepine receptors (CBR; [4], [5]. Subsequently, DBI was found to interact also with peripheral-type benzodiazepine receptors, now called translocator protein [6]. More recently, it has been shown that DBI-derived peptides can also activate a metabotropic receptor coupled either to adenylyl cyclase (AC) or to phospholipase C (PLC) [7]–[10]. The sequence of ODN has been well conserved during evolution [11], and data suggest that this peptide is involved in the regulation of important biological functions such as the control of food intake, sleep, aggressiveness and anxiety [12]–[17].

In situ hybridization experiments have shown that, in the brain, the DBI gene is primarily expressed by glial cells [18], [19]. The occurrence of DBI-related peptides in various populations of astroglial cells has been confirmed by immunohistochemistry [20]–[22]. In vitro studies have shown that cultured rat astrocytes contain and release substantial amounts of endozepines and that endozepine secretion is modulated by neuroactive compounds including the neuroprotective peptide, pituitary adenylate cyclase-activating polypeptide (PACAP) [23], [24].

Reactive astrogliosis is a common feature in many kind of brain injuries – *i.e.* ischemia, trauma and neurodegenerative diseases – and activated glial cells are traditionally thought to exert detrimental effects by releasing pro-inflammatory compounds and by inhibiting neuron regeneration [25]–[27]. Nevertheless, several studies suggest that reactive astrocytes may also contribute to the defense of neurons against oxidative stress [27]–[29]. In particular, it is now established that astrocytes contain high levels of reactive oxygen species (ROS) scavenger molecules and antioxidant enzymes, which are not only involved in the protection of astroglial cells against the deleterious effects of ROS [30] but may also play a critical role for neuron survival [31]–[33]. Little was known however about the endogenous factors that contributed to astroglial cell survival. In this context, we have recently shown that, in cultured astrocytes, ODN exerts a protective effect upon the deleterious action of hydrogen peroxide (H_2_O_2_), which is responsible for cell death, by attenuating H_2_O_2_-induced ROS accumulation [34].

There is now evidence that ODN acts as an autocrine factor modulating astroglial cell activity, but the various effects of the peptide are mediated through different type of receptors. For instance, in cultured rat astrocytes, ODN increases intracellular calcium concentration through activation of a PLC-coupled receptor [7], [10] and stimulates cell proliferation through activation of CBR [35]. Currently, nothing is known regarding the mechanism by which ODN exerts its glioprotective action. The purpose of the present study was thus to investigate the type of receptor and the signaling cascade involved in the beneficial effect of ODN against oxidative stress-induced cell death.

## Results

### Involvement of the ODN Metabotropic Receptor in the Protective Effect of the Peptide Against H_2_O_2_-Induced Astroglial Cell Death

**Figure 1:**
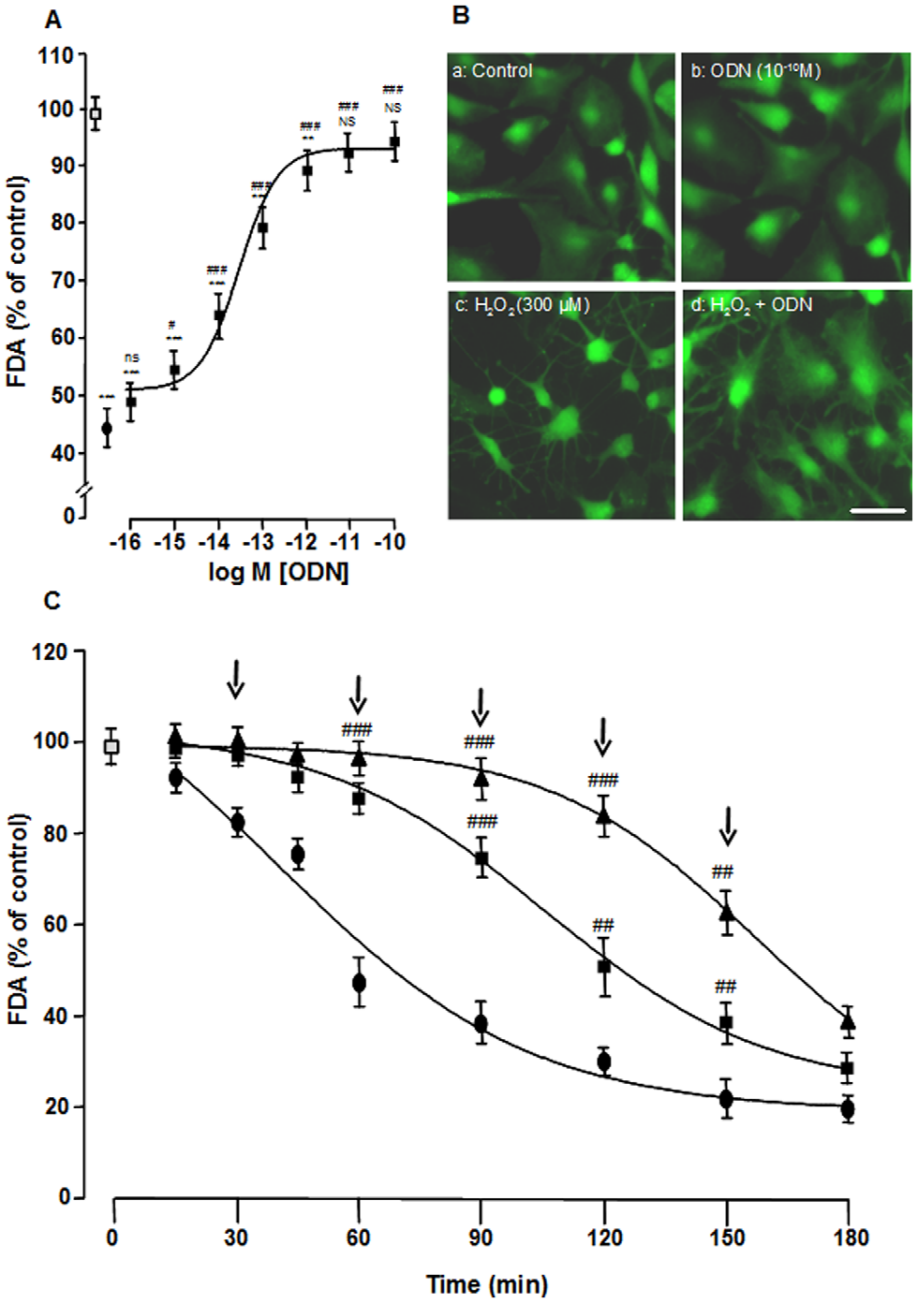
Protective effect of ODN on astroglial cell death induced by H_2_O_2_. (A) Cultured astrocytes were pre-incubated for 10 min in the absence or presence of graded concentrations of ODN (1 fM–1 nM) and then incubated for 1 h with medium alone (□) or with 300 µM H_2_O_2_ without (•) or with ODN (▪). Cell survival was quantified by measuring FDA fluorescence intensity, and the results are expressed as percentages of control. Each value is the mean (± SEM) of at least 12 different wells from three independent cultures. ANOVA followed by the Bonferroni's test: ** p<0.01; *** p<0.001; NS, not statistically different *vs.* control. ^#^ *p*<0.05; ^###^ *p*<0.01; ns, not statistically different *vs.* H_2_O_2_-treated cells. (B) Typical images illustrating the protective effect of ODN on H_2_O_2_-induced cell death. Cells were pre-incubated for 10 min in the absence (a, c) or presence of 0.1 nM ODN (b, d), and then incubated for 1 h with medium alone (a), ODN (b) or with 300 µM H_2_O_2_ without (c) or with ODN (d). Living astrocytes were labeled with calcein-AM (green fluorescence staining). Scale bar = 50 µm. (C) Time-course effect of ODN on H_2_O_2_-induced cell death. Cells were incubated for the indicated times with medium alone (□) or 300 µM H_2_O_2_ without (•) or with ODN (0.1 nM; ▪). After 30, 60, 90, 120 or 150 min of incubation, a refill of 0.1 nM ODN (arrows; ▴) was added in the culture medium. Cell survival was quantified by measuring FDA fluorescence intensity, and the results are expressed as percentages of control. Each value is the mean (± SEM) of at least 12 different wells from three independent cultures. ANOVA followed by the Bonferroni's test. ^##^ *p*<0.01; ^###^ *p*<0.001 *vs.* H_2_O_2_-treated cells.

We have previously shown that ODN, at a concentration of 0.1 nM, is able to reverse the effect of 300 µM H_2_O_2_-induced astrocyte cell death [34]. In order to determine the receptor involved in the protective effect of ODN, we have first examined the dose- and the time-course effect of the peptide on detrimental action of H_2_O_2_. The protective action of ODN was concentration-dependent for doses ranging from 1 fM to 0.1 nM (Figure 1A). The half-maximum effect was observed at a concentration of 0.04 pM and the maximum effect (∼95% survival) was obtained at a concentration of 0.1 nM. The protective effect of ODN on cultured astrocytes was also visualized by staining cells with calcein-AM. After a 1-h incubation in serum-free culture medium, in the absence or presence of 0.1 nM ODN, all astrocytes were alive, and calcein-labeled cells displayed a flat polygonal morphology (Figure 1Ba and 1Bb). Incubation with 300 µM H_2_O_2_ induced marked morphological changes, including shrinkage of cell bodies, retraction of processes and disappearance of calcein labeling (Figure 1Bc). Pretreatment of cells with ODN totally prevented the deleterious effects of H_2_O_2_ (Figure 1Bd).

Time-course experiment showed that ODN (0.1 nM) totally blocked H_2_O_2_-induced cell injury during the first 60 min (Figure 1C). Thereafter, the action of ODN declined and vanished 180 min after the onset of peptide administration. Addition of fresh doses of ODN (0.1 nM) after 30, 60, 90, 120 and 150 min of incubation prolonged the protective effect of the peptide at least for 150 min (Figure 1C).

**Figure 2:**
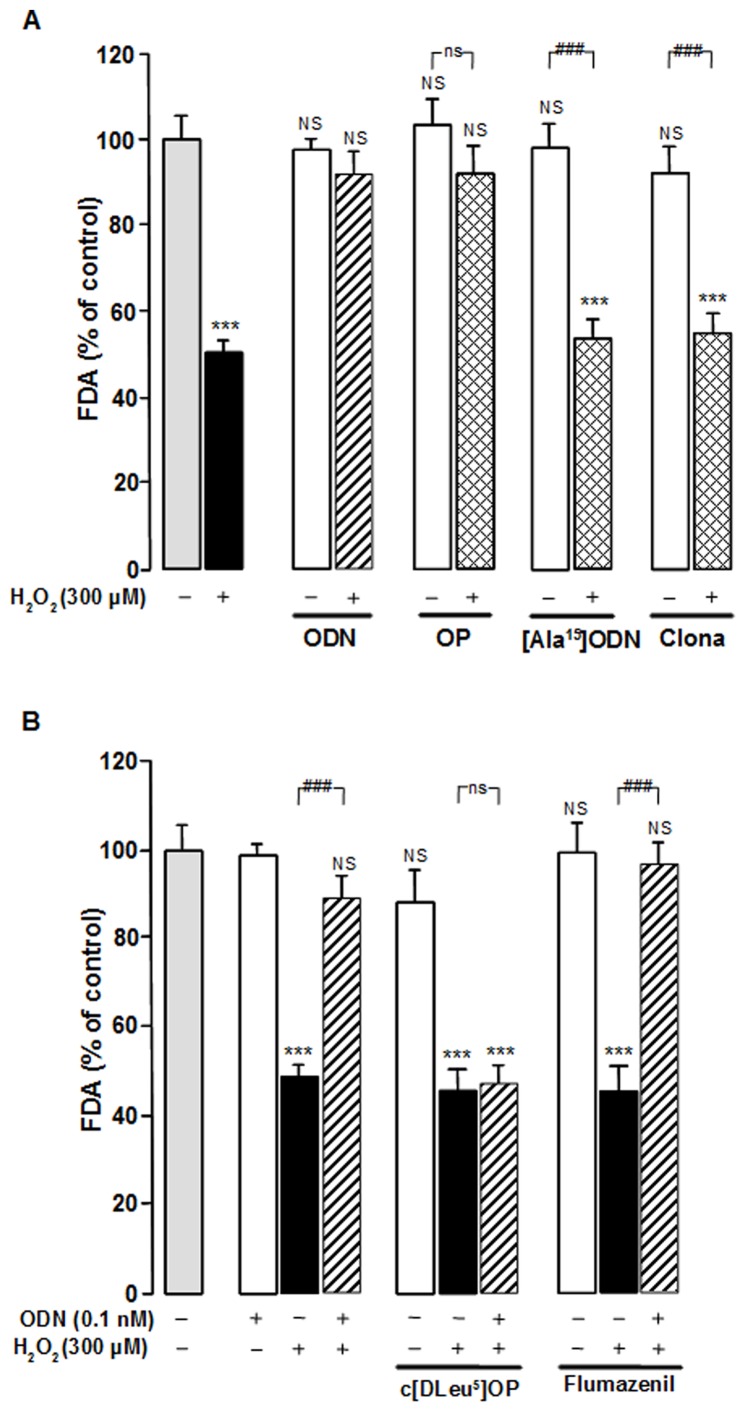
Pharmacological characterization of the receptor involved in the protective effect of ODN on astroglial cells. (A) Cultured astrocytes were pre-incubated for 30 min in the absence or presence of ODN (0.1 nM), the metabotropic receptor agonist OP (0.1 nM), the inactive ODN analog [Ala^15^]ODN (0.1 nM) or the specific CBR agonist clonazepam (Clona; 10 nM), and then incubated for 1 h with medium alone or with 300 µM H_2_O_2_ without or with receptor ligands. (B) Cells were pre-incubated for 30 min in the absence or presence of the metabotropic receptor antagonist cyclo_1–8_ [DLeu^5^] OP (c[DLeu^5^] OP; 1 µM) or the CBR antagonist flumazenil (1 µM), and then incubated for 1 h with medium alone or with 300 µM H_2_O_2_ without or with ODN (0.1 nM). Cell survival was quantified by measuring FDA fluorescence intensity, and the results are expressed as percentages of control. Each value is the mean (± SEM) of at least 12 different wells from three independent cultures. ANOVA followed by the Bonferroni's test. *** p<0.001; NS, not statistically different *vs.* control. ^###^ *p*<0.001; ns, not statistically different *vs.* H_2_O_2_-treated cells.

We next examined the effect of specific agonists and antagonists on H_2_O_2_-induced cell death. The protective effect of ODN was mimicked by the metabotropic receptor agonist octapeptide (OP; 0.1 nM) whereas its inactive analog, [Ala^15^]ODN (0.1 nM), was devoid of protective activity (Figure 2A). In addition, the specific central-type benzodiazepine receptor agonist clonazepam (10 nM) was unable to block the effect of H_2_O_2_. Pre-incubation of astrocytes for 30 min with the selective ODN metabotropic receptor antagonist, cyclo_1–8_[DLeu^5^]OP (1 µM), which had no effect by itself on cell survival, totally abolished the glioprotective action of ODN. In contrast, the central-type benzodiazepine receptor antagonist flumazenil (1 µM) did not affect the protective effect of ODN on H_2_O_2_-injured astrocytes (Figure 2B).

### The Glioprotective Effect of ODN Against H_2_O_2_-Induced Astrocyte Death is Mediated Through the PKA and MEK Pathways

**Figure 3:**
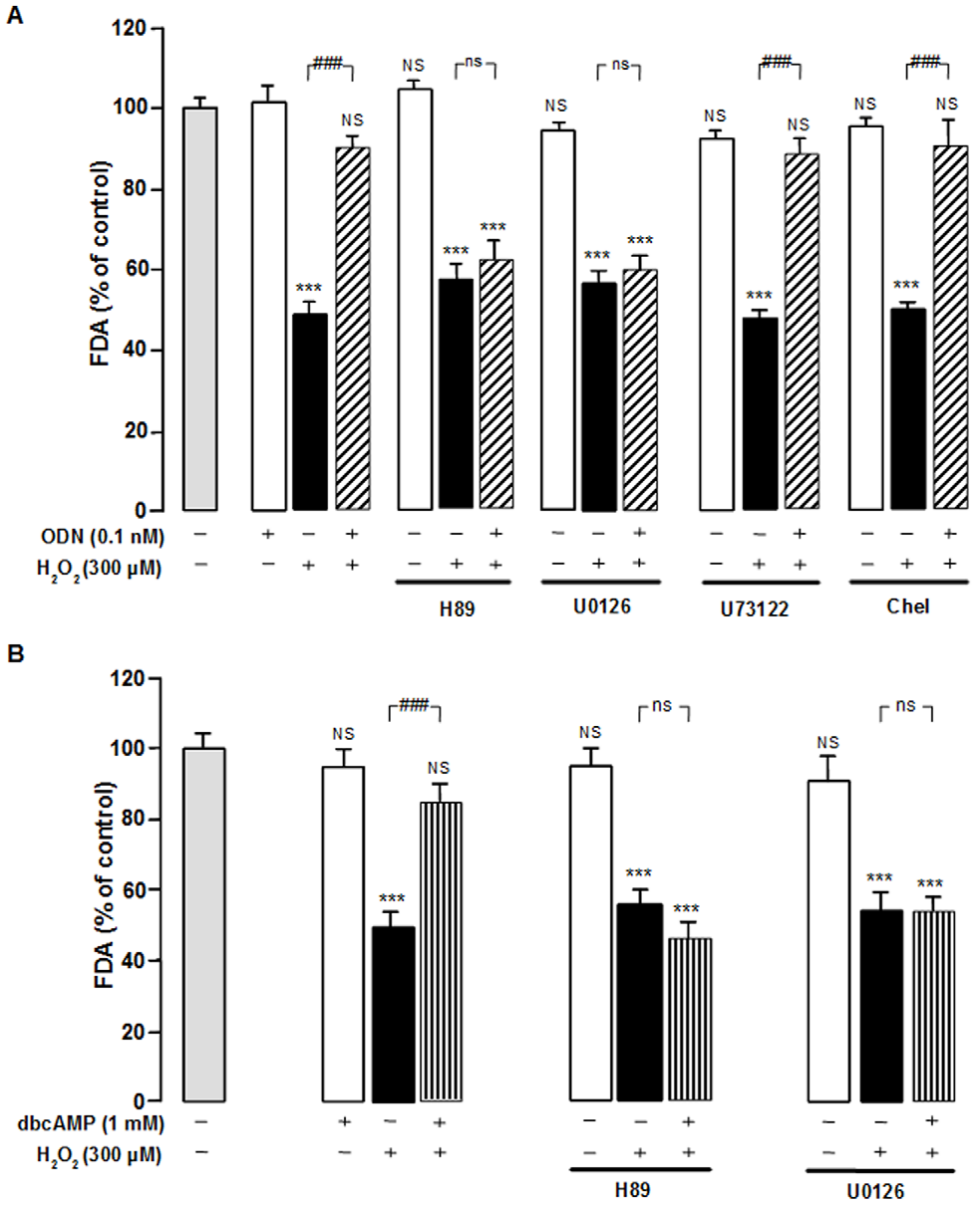
Identification of intracellular pathways involved in the protective effect of ODN on astroglial cells. (A) Cultured astrocytes were pre-incubated for 30 min in the absence or presence of H89 (20 µM), U0126 (20 µM), U73122 (1 µM) and chelerythrine (Chel; 1 µM) and then incubated for 1 h with medium alone, ODN (0.1 nM), or with 300 µM H_2_O_2_ without or with ODN (0.1 nM). (B) Cultured astrocytes were pre-incubated for 30 min in the absence or presence of the PKA inhibitor H89 (20 µM) and the ERK inhibitors U0126 (20 µM), and then incubated for 1 h with medium alone or with 300 µM H_2_O_2_ without or with dbcAMP (1 mM). Cell survival was quantified by measuring FDA fluorescence intensity, and the results are expressed as percentages of the control. Each value is the mean (± SEM) of at least of 12 different wells from three independent cultures. ANOVA followed by the Bonferroni's test. *** p<0.001; NS, not statistically different *vs.* control. ^###^ *p*<0.001; ns, not statistically different *vs.* H_2_O_2_-treated cells.

Incubation of astrocytes with the selective protein kinase A (PKA) inhibitor H89 (20 µM) or the mitogen-activated protein kinase kinase (MEK) inhibitor U0126 (20 µM) totally abrogated the protective action of ODN on astroglial cells (Figure 3A). In contrast, administration of the PLC inhibitor U73122 (10 µM) or the PKC inhibitor chelerythrine (0.1 µM) did not modify the protective effect of ODN on H_2_O_2_-evoked astrocyte death (Figure 3A). Incubation of astrocytes with the cell permeant cAMP analog dbcAMP (1 mM) mimicked the protective effect of ODN on H_2_O_2_-evoked cell death (Figure 3B). The effect of dbcAMP on astroglial cell survival was also suppressed by H89 and U0126 (Figure 3B).

### ODN Stimulates Adenylyl Cyclase Activity and ERK Phosphorylation in Astrocytes

**Figure 4:**
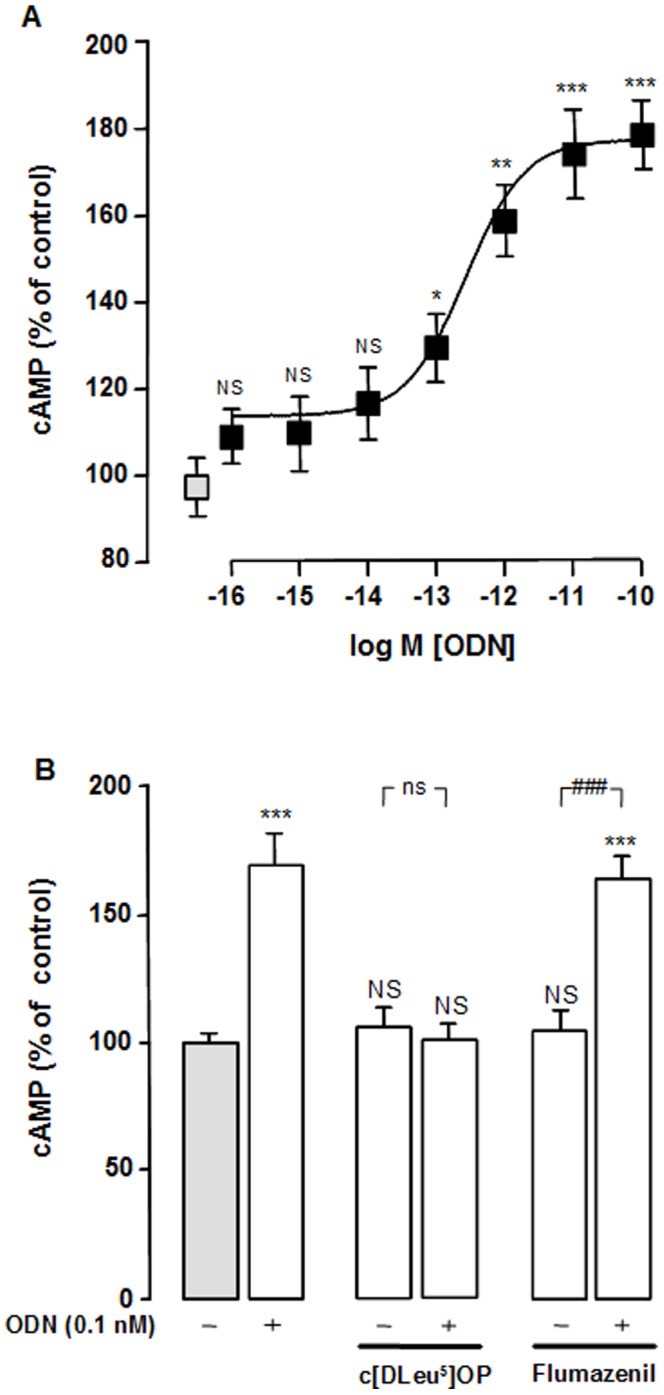
Effect of ODN on cAMP formation in astroglial cells. (A) Cultured astrocytes were pre-incubated for 30 min in the presence of 0.1 mM IBMX, and then incubated for 10 min with graded concentrations of ODN (1 fM–10 nM). (B) Cultured astrocytes were pre-incubated for 30 min with 0.1 mM IBMX in the absence or presence of cyclo_1–8_ [DLeu^5^] OP (c[DLeu^5^] OP; 1 µM) or flumazenil (1 µM), and then incubated for 10 min with medium alone or with ODN (0.1 nM). The results are expressed as percentages of control. cAMP production was quantified by radioimmunoassay, and the results are expressed as percentages of control. Each value is the mean (± SEM) of at least 15 different wells from three independent cultures. ANOVA followed by the Bonferroni's test. * p<0.05; ** p<0.01; *** p<0.001; NS, not statistically different *vs.* control. ^###^ *p*<0.001; ns, not statistically different *vs.* cells incubated with antagonists.

We next examined the coupling of the ODN metabotropic receptor to PKA and MEK pathways in rat astrocytes. Incubation of cells with graded concentrations of ODN (1 fM to 0.1 nM) induced a dose-dependent increase of cAMP production (Figure 4A). The half-maximum effect was observed at a concentration of 0.3 pM and the maximum stimulation of cAMP formation (179% over control; *p*<0.001) was obtained at a dose of 10 pM. Addition of the metabotropic receptor antagonist cyclo_1–8_[DLeu^5^]OP (1 µM) in the culture medium had no effect on cAMP production by itself, but totally suppressed the stimulatory action of ODN on cAMP formation. In contrast, flumazenil (1 µM) did not affect the increase of cAMP induced by ODN (Figure 4B).

**Figure 5:**
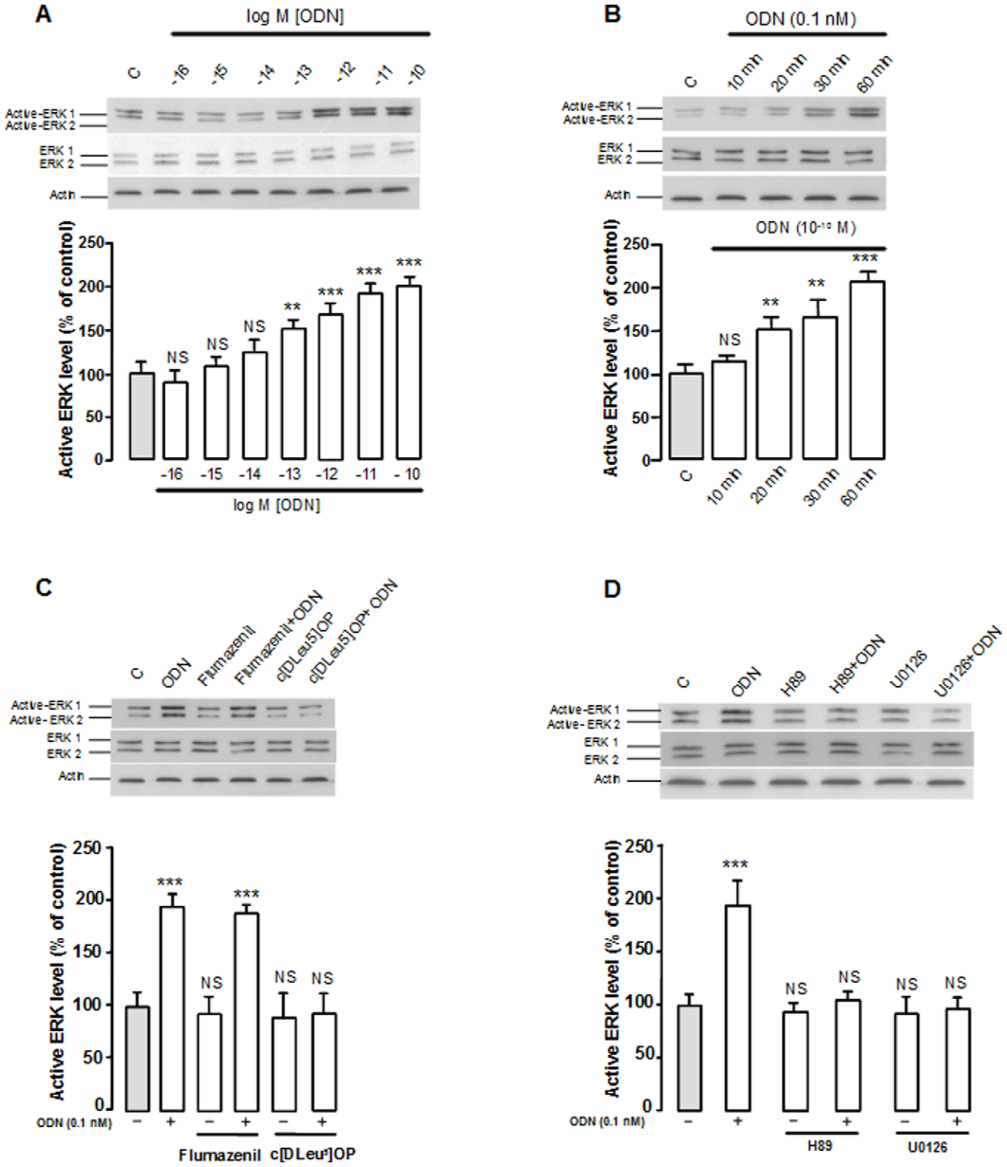
Effect of ODN on ERK phosphorylation in astroglial cells. (A–D) Cultured astrocytes were incubated with medium alone, with graded concentration of ODN (1 fM–1 nM; A) for 1 h, with 0.1 nM ODN for the times indicated (B), or with ODN (0.1 nM) in the absence or presence of flumazenil (1 µM; C), cyclo_1–8_ [DLeu^5^] OP (c[DLeu^5^] OP;1 µM; C), H89 (20 µM; D) or U0126 (20 µM; D) for 1 h. Active ERK1 and ERK2 were detected by Western blotting using antibodies against phosphorylated ERK and quantified by using total ERK and actin as internal controls. The results are expressed as percentages of control. Each value represents the mean (± SEM) of 12 different wells from three independent cultures. ANOVA followed by the Bonferroni's test. ** *p*<0.01; *** *p*<0.001; NS, not statistically different *vs.* control.

Similarly, Western blotting analysis revealed that graded concentrations of ODN (1 fM to 0.1 nM) induced a concentration-related stimulation of ERK phosphorylation in cultured rat astrocytes (Figure 5A). The time-course effect of ODN showed a detectable increase of ERK phosphorylation within 20 min and reached 206% after 60 min of treatment (Figure 5B). Addition of cyclo_1–8_[DLeu^5^]OP (1 µM) in the culture medium had no effect on ERK phosphorylation but totally suppressed the stimulatory effect of ODN (Figure 5C). In contrast, flumazenil (1 µM) did not affect ODN-induced ERK phosphorylation (Figure 5C). The effect of ODN on ERK phosphorylation was suppressed by the PKA inhibitor H89 as well as the MEK inhibitor U0126 (Figure 5D).

### ODN Exerts its Protective Effect Through the Intrinsic Mitochondrial Pathway

**Figure 6:**
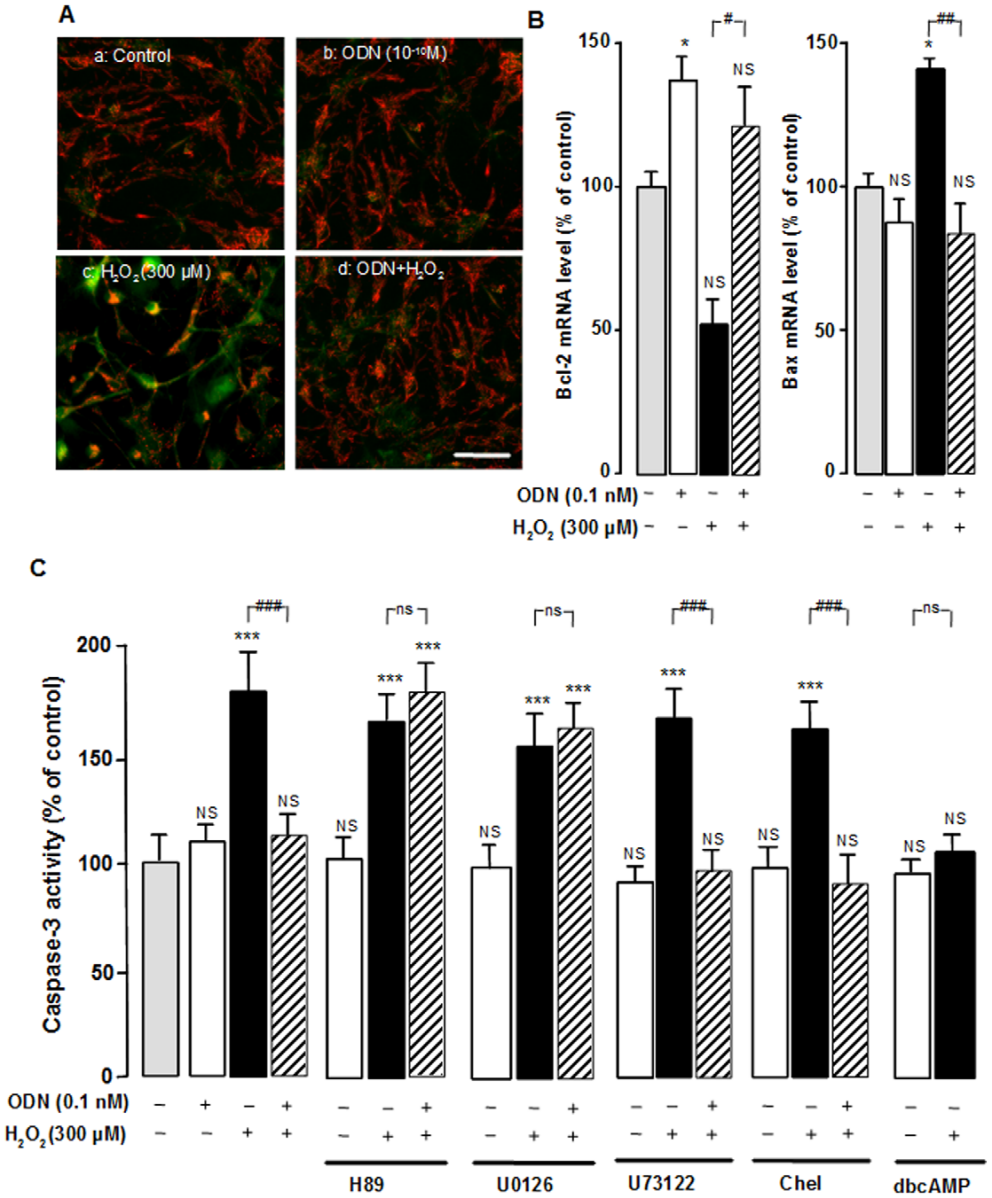
Involvement of the mitochondrial intrinsic pathway in the protective effect of ODN on astroglial cells. (A) Cultured astrocytes were pre-incubated for 10 min in the absence (a, c) or presence of 0.1 nM ODN (b, d), and then incubated for 1 h with medium alone (a), ODN (b) or with 300 µM H_2_O_2_ without (c) or with ODN (d). Aggregated (red signal) and monomeric (green signal) fluorescent JC-1 dye revealed active and inactive mitochondria, respectively. Scale bar, 50 µm. (B) Cultured astrocytes were pre-incubated for 10 min in the absence or presence of 0.1 nM ODN, and then incubated for 1 h with medium alone, ODN or with 300 µM H_2_O_2_ without or with ODN. Bcl-2 and Bax mRNA levels were quantified by RT-PCR. Results are expressed as percentages of control. Each value is the mean (± SEM) of 6 different wells from three independent cultures. ANOVA followed by the Bonferroni's test. * *p*<0.05; NS, not statistically different *vs.* control. ^#^ *p*<0.05; ^##^ *p*<0.01 *vs.* H_2_O_2_-treated cells. (C) Cultured astrocytes were pre-incubated for 30 min in the absence or presence of H89 (20 µM), U0126 (20 µM), U73122 (1 µM) or chelerythrine (Chel; 1 µM) and then incubated for 1 h with medium alone, ODN (0.1 nM), dbcAMP (1 mM) or with 300 µM H_2_O_2_ without or with ODN (0.1 nM) or dbcAMP (1 mM). Caspase-3 activity was quantified by measuring the fluorescence of caspase substrate, Z-DEVD-Rhodamine 110, and the results are expressed as percentages of control. Each value is the mean (± SEM) of at least 12 different wells from three independent cultures. ANOVA followed by the Bonferroni's test. *** p<0.001; NS, not statistically different *vs.* control. ^###^ *p*<0.001; ns, not statistically different *vs.* H_2_O_2_-treated cells.

The effect of ODN on the mitochondria integrity was first examined by visualizing the membrane potential using the fluorescent ratiometric probe JC-1. Control and ODN-treated astrocytes exhibited many active mitochondria (red fluorescence) located in the cell body and along their processes (Figure 6Aa and 6Ab). In contrast, treatment with H_2_O_2_ (300 µM) resulted in a marked decrease of the red signal in the mitochondria with a strong green fluorescent signal in the cell bodies, indicating that mitochondrial integrity was severely altered (Figure 6Ac). Pretreatment of cells with ODN suppressed this deleterious effect of H_2_O_2_ on the mitochondrial membrane potential with only few cells labeled in green.

The effect of ODN on the expression of the anti-apoptotic gene Bcl-2 and the pro-apoptotic gene Bax was also studied by quantitive reverse transcription-polymerase chain reaction (RT-PCR). Exposure of cultured astrocytes to ODN (0.1 nM) induced an increase of Bcl-2 mRNA levels by itself and suppressed the inhibitory effect of H_2_O_2_ on Bcl-2 expression (Figure 6B). In addition, ODN which had no effect on Bax mRNA levels totally prevented the increase of Bax mRNA levels induced by H_2_O_2_ (Figure 6B).

Finally the transduction pathways involved in the inhibitory action of ODN on H_2_O_2_-induced caspase-3 activity were characterized. H89 and U0126 abrogated the inhibitory effect of ODN on H_2_O_2_-induced caspase-3 activity. In contrast, neither U73122 nor chelerythrine could suppress the effect of ODN. Addition of dbcAMP (1 mM) in the culture medium mimicked the effect ODN on H_2_O_2_-evoked caspase-3 activation (Figure 6C).

### ODN Prevents H_2_O_2_-Induced Glutathione Depletion

**Figure 7:**
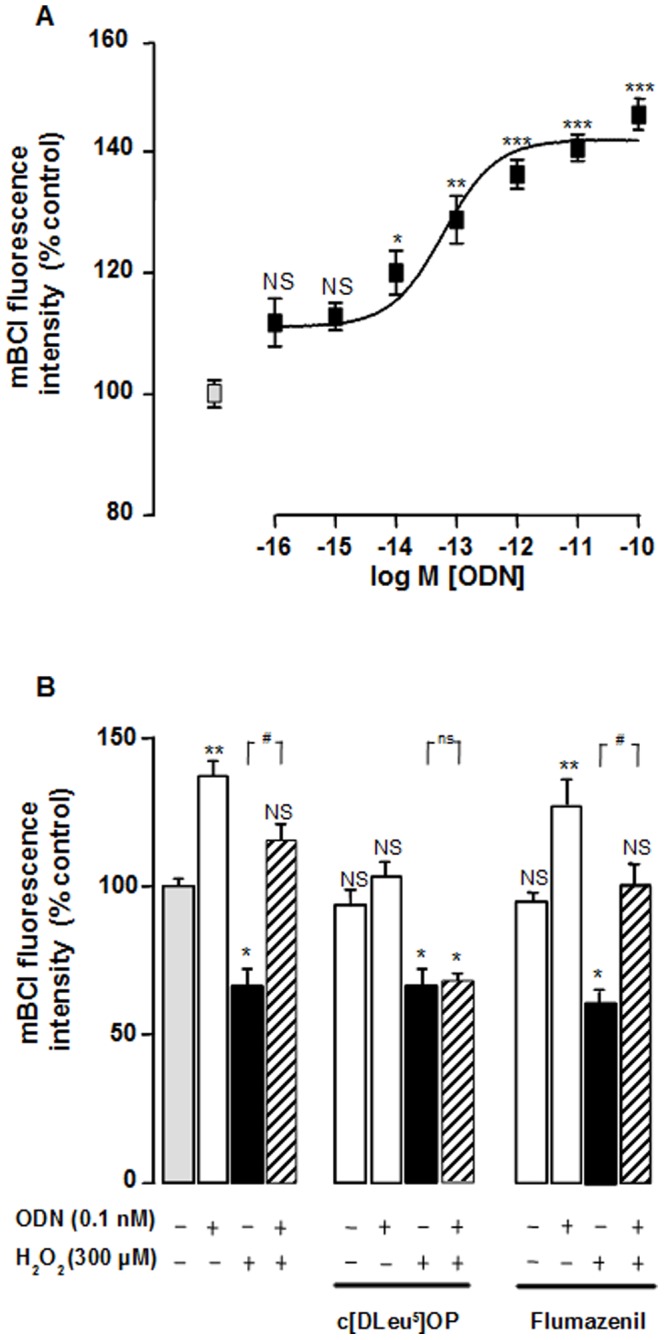
Effects of ODN on H_2_O_2_-induced intracellular depletion of glutathione. **C**ultured astrocytes were incubated with medium alone or with graded concentration of ODN (1 fM–0.1 nM) for 1 h. (B) Cells were pre-incubated for 30 min in the absence or presence of the metabotropic receptor antagonist cyclo_1–8_ [DLeu^5^] OP (c[DLeu^5^] OP; 1 µM) or the CBR antagonist flumazenil (1 µM), and then incubated for 1 h with medium alone, ODN (0.1 nM), or 300 µM H_2_O_2_ without or with ODN (0.1 nM). Cellular glutathione content was quantified by measurement of mBCl fluorescence, and the results are expressed as percentages of fluorescence of control. Each value is the mean (± SEM) of at least 12 different wells from three independent cultures. ANOVA followed by the Bonferroni's test. *p<0.05; ** p<0.01; ***p<0.001; NS, not statistically different vs control. *^#^p*<0.05; ns, not statistically vs H_2_O_2_-treated cells.

Since glutathione (GSH) is the most abundant thiol tripeptide for ROS scavenger in cells, we examined the effect of ODN on the content of intracellular GSH by using mBCl, a probe which forms a fluorescent compound when conjugated with GSH. Graded concentrations of ODN (1 fM to 0.1 nM) induced a dose-related increase in mBCl fluorescence intensity (Figure 7A), and pretreatment of cells with ODN (0.1 nM) prevented the depletion of mBCl fluorescence induced by H_2_O_2_ (300 µM) (Figure 7B). Addition of cyclo_1–8_[DLeu^5^]OP (1 µM) in the culture medium had no effect on mBCl fluorescence intensity but totally abolished ODN-stimulated GSH formation. Cyclo_1–8_[DLeu^5^]OP also suppressed the inhibitory effect of ODN on H_2_O_2_-evoked GSH content (Figure 7B). In contrast, flumazenil (1 µM) did not impair the effect of ODN on glutathione production neither in control nor in H_2_O_2_-treated cells (Figure 7B).

## Discussion

It has been previously reported that oxidative stress causes apoptosis in various cell types, including glial cells [30]. We have recently found that the endozepine ODN protects astrocytes upon H_2_O_2_ injury [34]. The present study reveals that the protective action of ODN against H_2_O_2_-induced astrocyte apoptosis is mediated through activation of a metabotropic receptor coupled to AC. Activation of the AC/PKA pathway leads to an increase of ERK phosphorylation which, in turn, is responsible for inhibition of the H_2_O_2_-induced increase of caspase-3 activity.

In agreement with previous data we found that the gliopeptide ODN, at very low doses, exerts a protective effect against oxidative stress on astrocytes. Visualization of living cells by calcein-AM staining revealed that the cytotoxic effect of H_2_O_2_ was associated with modifications of astrocyte morphology, such as cell shrinkage and appearance of long thin processes that were suggestive of apoptotic cell death. These morphological changes were also prevented by addition of subnanomolar concentrations of ODN in the medium. The time-course effect of ODN showed that the peptide delayed by approximately 1 h H_2_O_2_-induced cell death. The protective action of ODN was prolonged by peptide recharges in the medium, suggesting that breakdown of ODN occurred in our culture conditions. In agreement with this hypothesis, previous kinetic studies have revealed that ODN provokes a transient stimulation of superoxide dismutase and catalase activities in cultured astrocytes, and that refills of ODN prolong its stimulatory effects [34]. Altogether, these data suggest that ODN is sensitive to proteolytic enzymes released by cultured astrocytes [36]–[38]. However, it cannot be excluded that the decrease in the responses of astrocytes to ODN might in part be due to desensitization of the receptors. As a matter of fact in spite of recharges, the protective effect of ODN and its effects on superoxide dismutase and catalase activites [34] totally disappeared after 1–2 h of incubation.

There is now clear evidence that astroglial cells, like neurons, are vulnerable to oxidative stress [30], [39]–[41], and that apoptosis of astrocytes is observed in brain injuries caused by trauma, ischemia and neurodegenerative diseases [27], [42], [43]. The quantity of ODN needed to delay the deleterious effects of H_2_O_2_ on cultured astrocytes is similar to that measured in culture medium of astrocytes treated with PACAP, a glio/neuroprotective neuropeptide [24], [44], [45] whose gene expression is transitory increased after cerebral injury [46], [47]. This observation suggests that, under mild or moderate insults, endozepines, through a paracrine mode of action, could protect astroglial cells against oxidative stress-induced toxicity.

Previous studies have shown that, in astrocytes, ODN can interact with either CBR, associated with the GABA_A_ receptor complex [35] or with a metabotropic receptor positively coupled to PLC [10], [48]. It has also been found that a cyclic analog of ODN, cyclo_1–8_[DLeu^5^]OP, exhibits potent antagonistic activities on ODN-induced polyphosphoinositide turnover increase and intracellular calcium mobilization in rat astrocytes [7]. Here, we provide the first evidence that the astroglial metabotropic ODN receptor is also coupled to AC and that this metabotropic receptor is involved in the effect of ODN on H_2_O_2_-evoked astrocyte cell death : *i)* subnanomolar concentrations of ODN provoked a dose-dependent increase in cAMP production, *ii)* the metabotropic ODN receptor antagonist cyclo_1–8_[DLeu^5^]OP totally abolished the effect of ODN on both cAMP production and cell survival, and *iii)* in contrast, the selective CBR antagonist flumazenil did not impair these effects of ODN.

We next investigated the signaling cascade involved in the glioprotective effect of ODN. The cell-permeant cAMP analog dbcAMP prevented cultured astrocytes from H_2_O_2_ toxicity while the PKA inhibitor H89 abrogated the protective effect of ODN against H_2_O_2_-induced cell death. In contrast, neither the PKC inhibitor chelerythrine nor the PLC inhibitor U73122 had any effect on ODN-evoked astroglial cell survival. These observations thus indicate that the protective action of ODN against oxidative stress-induced cell death can be ascribed specifically to activation of the AC/PKA signaling pathway. The involvement of the AC cascade in the protective effect of ODN against H_2_O_2_ is in agreement with data showing that Gs protein activation, which stimulates AC activity and cAMP formation, protects hippocampal HT22 neurons from H_2_O_2_ toxicity [49]. There is now increasing evidence that blockage of the MAP-kinase signaling cascade induces apoptosis in various cell types, including astrocytes [50]. For instance, it has been demonstrated that protection of glial cells against H_2_O_2_ or ischemia injury requires MEK phosphorylation and ERK signaling cascade activation [42], [51]. Moreover, alteration of ERK activity in these cells leads to an accumulation of ROS and subsequent apoptosis due to oxidative stress [42], [52]. Consistent with these observations, the MEK blocker U0126 abrogated the effect of ODN on H_2_O_2_-induced cell death and suppressed the stimulatory action of ODN on ERK phosphorylation. In addition, ODN-induced phosphorylation of ERK was abolished by the metabotropic receptor antagonist cyclo_1–8_[DLeu^5^]OP as well as the PKA inhibitor H89, indicating that activation of the ODN receptor leads to an increase in PKA activity which is responsible for ERK phosphorylation, and thus protection of astrocytes from the deleterious effect of H_2_O_2_. The induction of ERK phosphorylation by PKA is consistent with data indicating that activation of the ERK cascade may be cAMP-dependent. In particular, it has been reported that the neuropeptide PACAP promotes astrocyte proliferation [53] and differentiation [54] through ERK phosphorylation via a cAMP-dependent pathway.

It has been previously shown that ODN is able to prevent H_2_O_2_-induced alteration of mitochondrial integrity in cultured astrocytes [34] and it is well known that mitochondrial membrane permeability is under the control of pro- and anti-apoptotic factors that belong to the Bcl-2 family [55], [56]. In cultured astrocytes, H_2_O_2_ exerts opposite effects on the expression of Bax, a pro-apoptotic member of the Bcl-2 family, and Bcl-2, an anti-apoptotic factor [57]. The observation that ODN stimulated Bcl-2 expression and totally suppressed the increase of Bax expression induced by H_2_O_2_ thus indicates that the peptide controls the balance between the pro- and anti-apoptotic factors Bax and Bcl-2.

Activation of caspase-3 plays a prominent role in H_2_O_2_-induced apoptosis of astrocytes [57], [58] and we have previously demonstrated that subnanomolar concentrations of ODN inhibit H_2_O_2_-induced caspase-3 activation in cultured astrocytes [34]. Here, we show that treatment of cells with H89 and U0126 abrogates the inhibitory action of ODN on H_2_O_2_-evoked caspase-3 activation while U73122 and chelerythrine have no effect. These data demonstrate that ODN-induced inhibition of caspase-3 activity in astrocytes is mediated through activation of both PKA and ERK-type MAP kinase transduction pathways. The fact that the cell survival-promoting effect of ODN is also mediated through an AC/PKA/MAP kinase-dependent mechanism, confirms that the glioprotective action of the peptide involves a reduction of caspase 3 activity. The activation of caspases leading to apoptosis is mediated through two distinct pathways, the extrinsic pathway and the intrinsic pathway that involves the participation of the mitochondria. The present results indicate that the action of ODN on the Bax/Bcl-2 balance is in favor of the anti-apoptotic factor, which is responsible in turn for the beneficial effect of ODN on mitochondrial integrity. In addition, ODN is able to increase, through activation of the metabotropic receptor, the level of the ROS scavenger GSH in astrocytes. The mechanism involved in this effect is currently unknown. However, it has been shown that an over-expression of Bcl-2 in PC12 cells is responsible for an increase of GSH level [59]. Thus, these results suggest that the protective effect of ODN on astrocytes is attributable to both an inhibition of caspase-3 activity through the intrinsic pathway, and a reduction of the production of ROS.

Several lines of evidence suggest that the endozepine ODN might play a role in the development of neurodegenerative disorders. The concentration of endozepines is elevated in the cerebral spinal fluid of patients with Alzheimer's and Parkinson's diseases [60] and it has been reported that β-amyloid peptide, the major constituent of senile plaques, induces endozepine biosynthesis and release from cultured astrocytes [61], [62]. Moreover, ODN exerts a proliferative effect on some cell types, notably astroglial cells [35], [62], suggesting that over-production of ODN may contribute to astrocyte proliferation observed in various neurodegenerative diseases. Besides, the present data indicate that ODN prevents H_2_O_2_-induced astrocyte apoptosis which is in agreement with a study showing that DBI silencing with siRNA leads to growth arrest and apoptosis in various mammalian cell lines [63]. Altogether, these data suggest that, in moderate brain injury, the endozepine ODN might exert a neuroprotective effect. Thus, up-regulation of endozepine production by astroglial cells might be a therapeutic option to reduce neuronal cell death in neurodegenerative diseases, trauma or stroke.

**Figure 8:**
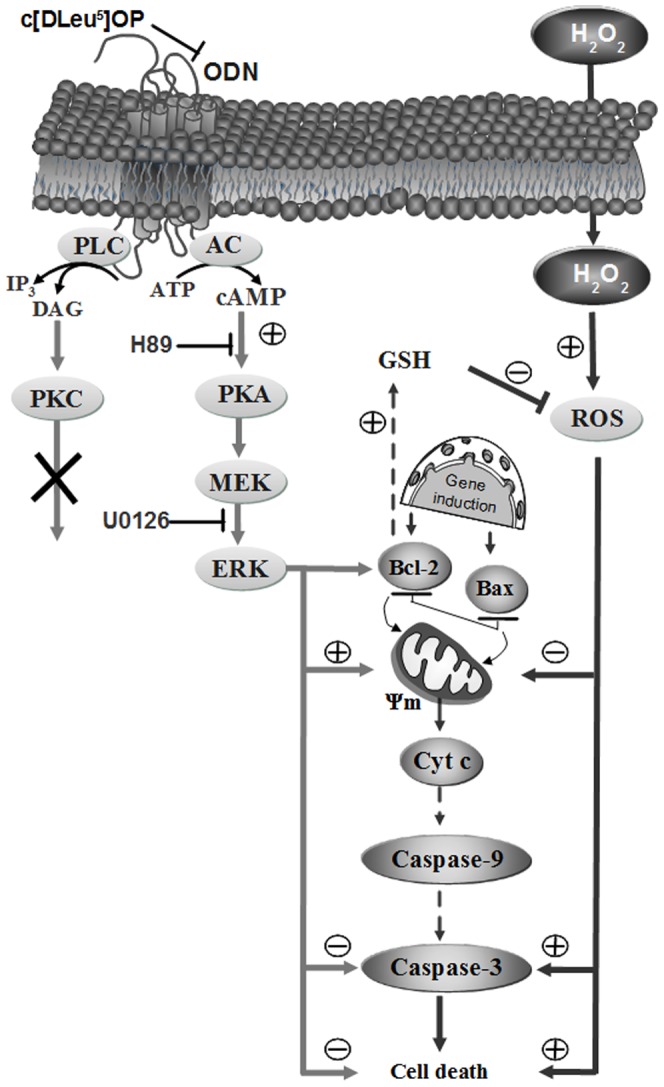
Schematic representation of the signaling pathways likely involved in the protective effect of ODN against H_2_O_2_-induced astroglial cell apoptosis. ODN activates both adenylyl cyclase (AC) and phospholipase C (PLC) in astrocytes, however only the cAMP-dependent protein kinase A (PKA) pathway is involved in the effect of ODN on cell survival. ODN stimulates phosphorylation of extracellular regulated kinase (ERK) in a PKA-dependent manner. Downstream, ODN activates the expression of the anti-apoptotic gene Bcl-2, stimulates glutathione (GSH) formation and abolishes H_2_O_2_-induced decrease of mitochondrial potential (Ψm) via the formation of highly reactive oxygen species (ROS), the inhibition of Bcl-2 and the stimulation of the pro-apoptotic gene Bax. Finally, ODN prevents H_2_O_2_-evoked activation of caspase-3 leading to astrocyte death. Cyt c, cytochrome c; DAG, diacylglycerol; IP_3_, inositol trisphosphate; H89, protein kinase A inhibitor; MEK, mitogen-activated protein kinase kinase; PKC, protein kinase C; U0126, MAP kinase kinase inhibitor. +, activation; –, inhibition.

In conclusion, the present study has demonstrated that ODN, acting through a metabotropic receptor sensitive to the cyclo_1–8_[DLeu^5^]OP antagonist, exerts a potent protective effect against apoptosis induced by oxidative stress in astrocytes. The anti-apoptotic effect of ODN can be ascribed to stimulation of the AC/PKA/ERK-kinase transduction pathway, which modulates the Bax/Bcl-2 balance in favor of an anti-apoptotic activity leading to an inhibition of H_2_O_2_-induced caspase-3 through the intrinsic apoptotic mitochondrial pathway, and by increasing GSH production, which in turn attenuates ROS formation (Figure 8).

## Materials and Methods

### Ethics Statement

The experiments of the current project have been made in accordance with American Veterinary Medical Association. Approval for these experiments was obtained from the Medical Ethical Committee For the Care and Use of Laboratory Animals of Pasteur Institut of Tunis. Approval Nu FST/LNFP/Pro 152012.

### Reagents

Dulbecco's modified Eagle's medium (DMEM), F12 culture medium, D(+)-glucose, L-glutamine, *N*-2-hydroxyethylpiperazine-*N*-2-ethane sulfonic acid (HEPES), fetal bovine serum (FBS), the antibiotic-antimycotic solution and trypsin-EDTA were obtained from Gibco (Invitrogen, Grand Island NY, USA). N^6^,2′-O-dibutyryladenosine 3′,5′ cyclic monophosphate 5 (dbcAMP), Chelerythrine, H89, isobutylmetylxanthine (IBMX), U73122, trichloroacetic acid (TCA), fluorescein diacetate-acetoxymethyl (FDA-AM), Triton X-100 and insulin were purchased from Sigma Aldrich (St. Louis, MO, USA). The probe JC-1 and calcein-AM were obtained from Molecular Probes (Eugene, Oregon, USA). Fluorometric assay reagent for caspase-3 was supplied by Promega (Charbonnière, France). U0126 was from Calbiochem (San Diego, CA, USA). cAMP radioimmunoassay (RIA) kit (IRC 118) was from Izotope (Budapest, Hungarian). Clonazepam and flumazenil was a generous gift from Hoffmann-La Roche (Basel, Switzerland). Rat ODN, OP, [Ala^15^]ODN and the ODN antagonist (cyclo_1–8_[DLeu^5^]OP) were synthesized by using the standard Fmoc procedure, as previously described [7].

### Cell Culture

Secondary cultures of rat cortical astrocytes were prepared as previously described [64]with minor modifications. Briefly, cerebral hemispheres from newborn Wistar rats were collected in DMEM/F12 (2∶1; v/v) culture medium supplemented with 2 mM L-glutamine, 1‰ insulin, 5 mM HEPES, 0.4% glucose and 1% of the antibiotic-antimycotic solution. The tissues were dissociated mechanically with a syringe equipped with a 1-mm gauge needle, and filtered through a 100-µm sieve (Falcon, Franklin Lakes, NJ, USA). Dissociated cells were resuspended in culture medium supplemented with 10% FBS, plated in 175-cm^2^ flasks (Greiner Bio-one GmbH, Frickenhausen, Germany) and incubated at 37°C in a 5% CO_2_/95% O_2_ atmosphere. When cultures were confluent, astrocytes were isolated by shaking overnight the flasks on an orbital agitator. Adhesive cells were detached by trypsination and preplated for 5 min to discard contaminating microglial cells. Then, the non-adhering astrocytes were harvested and plated on 35-mm hydrophilic surface Petri dishes at a density of 3×10^5^ cells/ml. For measurement of cell survival, caspase-3 activity and mitochondrial activity, cells were plated in hydrophilic surface 24-well plates at a density of 8×104 cells/ml. The cells were incubated at 37°C in a humid atmosphere (5% CO_2_). After 5 days (DIV5), more than 99% of the cells were labeled with antibodies against glial fibrillary acidic protein [65]. All experiments were performed on 5- to 7-day-old secondary cultures.

### Measurement of Cell Survival

Cultured cells were incubated at 37°C with fresh serum-free culture medium in the absence or presence of H_2_O_2_ and/or ODN for 1 h. Cells were then incubated for 10 min at 37°C with 0.3 µg/ml calcein-AM (producing green fluorescence in living cells), rinsed twice with culture medium without probe and examined on an inverted microscope (Leica, Heidelberg, Germany) equipped with a double pass filter.

For quantification of surviving astrocytes, cells were incubated for 8 min with 15 µg/ml FDA-AM, rinsed twice with phosphate-buffered saline (PBS) and lysed with a Tris/HCl solution containing 1% sodium dodecyl sulfate (SDS). Fluorescence was measured with excitation at 485 nm and emission at 538 nm, using a microplate reader (Bio-Tek FLx 800).

### Measurement of cAMP Production

Cultured cells were pre-incubated for 30 min in serum-free medium containing 0.1 mM IBMX to inhibit phosphodiesterases, and then incubated in the same solution, in the presence or absence of test substances. The incubation was stopped by removing the medium and adding 10% (w/v) ice-cold TCA. The cells were homogenized and centrifuged (14,000 *g*, 4°C, 10 min). The supernatant was washed three times with 0.5 ml water-saturated diethylether, dried by vacuum centrifugation, and reconstituted in RIA buffer (0.05 M sodium acetate, pH 5.8). The concentration of cAMP was measured by using a cAMP RIA kit. The pellets were used for measurement of protein concentration by the Bradford method.

### Measurement of intracellular glutathione content

Reduced GSH was measured by using the thiol-reactive probe mBCl. At the end of the treatment period, the culture medium was removed and the cells were incubated with the mBCl probe (40 µM) at 37°C for 30 min and then washed twice with PBS. Fluorescence intensity was measured with excitation at 360 nm and emission at 485 nm, using a microplate reader (Bio-Tek FLx 800).

### Measurement of Caspase-3 Activity

At the end of the incubation with test substances, cultured astrocytes were washed twice with PBS at 37°C, resuspended in DMEM (100 µl) and treated with fluorometric caspase-3 Apo-1 assay system. In brief, 100 µl of the cell suspension was incubated with 100 µl of kit buffer and caspase substrate in 96-well plates. Caspase-3 activity was calculated from the slope of the fluorescence measured every 15 min for 3 h with excitation at 485 nm and emission at 530 nm, and expressed as a percentage of the control.

### Quantitative RT-PCR Analysis

Cultured cells were incubated at 37°C for 1 h with fresh serum-free medium in the absence or presence of test substances. At the end of the incubation, the culture medium was removed and cells were washed twice with PBS. Total RNA was extracted by using Tri reagent (Sigma, St Quentin Fallavier, France) and purified using NucleoSpin RNA II kit (Macherey-Nagel, Hoerd, France). RT-PCR was performed on 15 ng of total cDNA with 1×SYBR Green universal PCR Master mix (Applied Biosystem, Courtaboeuf, France) containing dNTPs, MgCl_2_, AmpliTaq Gold DNA polymerase, 300 nM forward (5′-TGCAGAGGATGATTGCTGATGT-3′) and reverse (5′-CAGCTGCCACACGGAAGAA-3′) Bax primers or forward (5′-GGCTGGGATGCCTTTGTG-3′) and reverse (5′-CAGCCAGGAGAAATCAAACAGA-3′) Bcl-2 primers (300 nM, each; Proligo, Paris, France), using the ABI Prism 7000 sequence detection system (Applied Biosystem). The amount of Bax and Bcl-2 cDNA in each sample was calculated by the comparative threshold cycle (Ct) method and expressed as 2^exp(−ΔΔCt)^ using glyceraldehyde-3-phosphate dehydrogenase as an internal control.

### Western Blot Analysis

Cultured cells were incubated at 37°C for 1 h with fresh serum-free medium in the absence or presence of test substances. Total proteins were extracted with lysis buffer containing 1% Triton X-100, 50 mM Tris/HCl and 10 mM EDTA. After centrifugation (20,000 *g*, 4°C, 15 min), the proteins contained in the supernatant were precipitated by addition of ice-cold 10% TCA. The extract was centrifuged (15,000 *g*, 4°C, 15 min) and washed three times with alcohol/ether. The pellet was denatured (100°C, 5 min) in 50 mM Tris/HCl (pH 7.5) containing 20% glycerol, 0.7 M 2-mercaptoethanol, 0.004% bromophenol blue and 3% SDS, and then migrated on a 10% SDS-polyacrylamide gel electrophoresis (PAGE). After separation, proteins were electrically transferred onto nitrocellulose membrane (Amersham, Les Ulis, France). The membrane was incubated with a blocking solution at room temperature for 1 h, and then revealed with antibodies against actin (Santa Cruz Biotechnology, Santa Cruz, CA, USA), phospho ERK (Promega) and total ERK (Promega), using a chemiluminescence detection kit (ECL System, Amersham, Aylesbury, United Kingdom). Signal was quantified using an image analysis system (Biocom, Les Ulis, France).

### Mitochondrial Activity Analysis

Cells seeded into 24-well plates were subjected to various treatments, incubated in the presence of the JC-1 probe (3 µl/ml) at 37°C for 15 min and then washed twice with PBS. Mitochondrial integrity was assessed with the JC-1 probe. In healthy astrocytes, the normal membrane potential allows accumulation and aggregation of the lipophilic dye JC-1 into the mitochondria (red signal) whereas, in dead cells, the mitochondrial membrane potential collapses and the monomeric JC-1 remains in the cytosol (green signal). Images were acquired using an eclipse E-600 microscope (Nikon, Champigny-sur-Marne, France) equipped with a 3 CCD Sony DXC950 camera interfaced with the Visiolab computerized program (Biocom, Les Ulis, France).

### Statistical Analysis

Data are presented as the mean ± SEM from three independent experiments performed in quadruplicate or quintuplicate. Statistical analysis of the data was performed by using Student's *t* test and ANOVA, followed by Bonferroni's test. A *p* value of 0.05 or less was considered as statistically significant.
